# Microstructure and Mechanical Properties of Mg-Li/Al Dissimilar Joints via Dynamic Support Friction Stir Lap Welding

**DOI:** 10.3390/ma17122883

**Published:** 2024-06-13

**Authors:** Yisong Gao, Yingying Zuo, Huijie Liu, Dongrui Li, Xuanmo Li

**Affiliations:** State Key Laboratory of Advanced Welding and Joining, Harbin Institute of Technology, Harbin 150001, China

**Keywords:** Mg-Li/Al dissimilar joint, dynamic supporting friction stir welding, microstructure, interface formation, mechanical properties

## Abstract

In this study, two-mm-thick dual-phase LA103Z Mg-Li and 6061 Al alloys, known for their application in lightweight structural designs, were joined using dynamic support friction stir lap welding (DSFSLW). The microstructural evolution and mechanical properties of dissimilar joints were investigated at different welding speeds. The analysis revealed two distinct interfaces: the diffusion interface and the mixed interface. The diffusion interface, characterized by a pronounced diffusion zone, is formed under slower welding speeds. The diffusion zone height, the effective lap width, and the interface layer thickness decrease with increasing welding speed due to low plastic deformation capacity and weak interfacial reactions. Conversely, the mixed interface, associated with higher welding speeds, contained large Al fragments. The extremely high microhardness values (130.5 HV) can be ascribed to the formation of intermetallic compounds (IMCs) and strain-hardened Al fragments. Notably, the maximum shear strength achieved was 175 N/mm at a welding speed of 20 mm/min. The fracture behavior varied significantly with the interface type; the diffusion interface showed enhanced mechanical strength due to better intermetallic reactions and interlocking structures, while the mixed interface displayed more linear crack propagation due to weaker IMCs and the absence of hook structures. Fracture surface analysis indicates that fractures are more likely to propagate through the Al matrix and interface layers.

## 1. Introduction

In the relentless pursuit of enhanced fuel efficiency and reduced emissions, the aerospace and automotive industries have turned to lightweight materials to meet these objectives [1,2]. The demand for materials that offer a combination of low weight, high strength, and superior shielding properties to electromagnetics has driven the adoption of hybrid components that surpass the capabilities of traditional single materials. This need is particularly acute in sectors like automotive, aerospace, and marine applications, where performance and durability are paramount [3,4]. Among these, Mg and Al alloys stand out for their low density and high specific strength, which are pivotal for the construction of energy-efficient structures [5,6].

In particular, as the lightest structural metal material currently available, Mg-Li alloys exhibit a density ranging from 1.30 to 1.65 g/cm³ [7,8]. The incorporation of lithium can diminish the c/a ratio of hexagonal close-packed (HCP) Mg alloys to improve the activity of non-basal <a> slip and <c+a> slip mode, which in turn improves the plasticity of the material [9]. According to the Mg-Li binary phase diagram, when the content of Li in Mg is between 5.3 wt% and 10.7 wt%, the HCP (α-Mg) structure transits to a mixed structure comprising HCP (α-Mg) and body-centered cubic (BCC, β-Li) [10]. The BCC structure, possessing five independent slip systems, further augments the plastic deformation capacity of the materials [11]. Given the superior properties of Mg-Li alloys, the joining of Mg-Li alloys with Al alloys is not only desirable but also necessary for fabricating advanced hybrid structures [12]. Nevertheless, the addition of Li in Mg-Li alloys increases their sensitivity to heat input, rendering them prone to volatilization and ablation when exposed to high-energy-density heat sources. Furthermore, the disparity in physical and chemical properties, such as melting point and thermal expansion coefficient between Mg-Li and Al alloy, may lead to defects such as voids and hot cracks during fusion welding [13], which significantly pose challenges in achieving a robust and reliable joint.

Friction stir lap welding (FSLW), a solid-state welding process, has emerged as a viable solution for the challenges associated with dissimilar materials joining [14,15]. The low heat input and the formation of solid-phase bonds are particularly beneficial for joining materials with different thermal and mechanical properties [16,17,18]. Hence, much research on the FSW of Mg and Al alloys has been explored. Studies have emphasized the process parameters, microstructures, interfacial intermetallic compounds (IMCs), and mechanical performance of the joints. Ji et al. [19] observed the formation of sharp hook structures in AZ31Mg/6061Al alloy joints and posited that such structures could diminish joint mechanical properties. Hu et al. [20] reported that bulk IMCs formed at high rotation speed and low welding speed, while the interface interlocking was seriously weakened at low rotation speed and high welding speed. Wu et al. [21] utilized swing friction stir spot welding to achieve the joining of AA5083 and AZ31B alloy, noting that interfacial IMC formation within the joint resulted in extremely high microhardness (150–250 HV). Xu et al. [22] characterized the interfacial stress in AZ31B/5A06 FSW joints using electron backscatter diffraction. The results show that immense stress and a stress gradient across the Al and Al_3_Mg_2_ layers lead to crack initiation and propagation. To date, extensive research has been conducted on the FSW of AZ31 Mg alloy with various Al alloys, revealing the complexity of the process and the need for optimization of welding parameters to achieve robust joints. Nonetheless, there exists a need for more research concerning the FSLW of the emergent Mg-Li alloys with Al alloys.

Generally, the predominant failure mechanism in Mg/Al joints appears to be due to the excessive formation of IMCs. However, for dissimilar materials, the frictional heat generated by lower melting point alloys, such as Mg-Li alloy, is reduced, potentially leading to insufficient interfacial reactions. Additionally, the lack of plasticization of the high melting point material at the bottom of the lap configuration may lead to the formation of holes and tunnel defects [23].

To surmount the obstacles, some improved FSW methods, such as pulse current-assisted FSW [24] and innovative FSW tools [25], have been proposed to enhance plastic flow and augment the metallurgical bonding strength. The strategy of reducing back heat dissipation to control heat distribution, inhibit the formation of welding defects, and improve joint performance has been applied in Ti alloy FSW [26,27]. Furthermore, Du et al. [28] developed a technique termed dynamic support friction stir welding (DSFSW), which employs a small-diameter support shoulder in place of the traditional backing plate. This design reduces the support area, curbing rapid heat loss at the weld’s back and diminishing the temperature gradient across the thickness. The application of DSFSW has achieved a defect-free TA5 Ti alloy joint, enhancing its mechanical properties. Given its attributes, DSFSW is likely suitable for joining dissimilar materials. However, no such applications have been reported in the literature.

In conclusion, the formation and properties of Mg-Li/Al FSLW joints are governed by a complex interplay of material properties, welding parameters, and microstructural evolution. A thorough understanding of these factors is essential for the development of welding procedures that can fully exploit the potential of Mg-Li alloys in engineering applications. The present work aims to investigate the joint formation, interfacial characteristics, and fracture behavior of Mg-Li/Al joints produced by dynamic support friction stir lap welding (DSFSLW). Specifically, the study delves into the impact of welding speed on the joint morphology, interface layer thickness, and mechanical properties. The interfacial microstructure is analyzed to correlate the failure process with microstructural features. This study seeks to contribute to the understanding of Mg-Li/Al dissimilar alloys FSLW and provide insights into the development of effective joining techniques for dissimilar materials.

## 2. Materials and Experimental Procedure

### 2.1. Base Materials and Experimental Procedures

**Figure 1:**
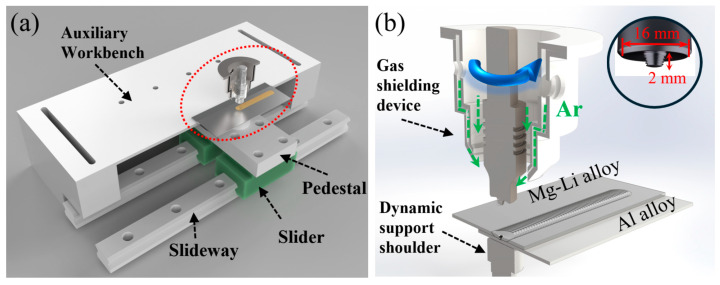
Schematic of DSFSLW of AA 6061 and LA103Z alloys: (**a**) 3D model drawing of the supporting component; (**b**) relative positions of welding component.

In the present work, AA6061-T6 Al alloy and LA103Z Mg-Li alloy plates in rolled states were employed as base materials (BM), conforming to the Chinese national standards [29,30]. Table 1 presents the nominal chemical compositions of these BMs. The plates were cut to dimensions of 225 mm × 65 mm × 2 mm and then milled and polished slightly to remove oxide and impurities, followed by degreasing with acetone. Figure 1 illustrates the schematic of DSFSLW, which includes the supporting system (composed of the slideways, slider, pedestal, and auxiliary workbench) and welding system (composed of the welding tool, dynamic support shoulder, and gas shielding device). The welding tool was fabricated by high-speed steel with a right-hand thread probe of 2 mm in length and a 10° concave shoulder of 16 mm in diameter. The dynamic support shoulder (DS-shoulder) with a diameter of 20 mm was employed to minimize heat dissipation from the weld back. The Al plate was placed on the DS-shoulder, and the Mg-Li plate was positioned on the Al plate in a lap configuration. The dimension of overlapping is 40 mm. Considering the oxidation of Mg-Li alloys, a gas shielding device with a continuous flow (20 L/min) of argon gas was employed during the welding process. The DSFSLW experiments were carried out with a displacement-control welding machine, with a shoulder plunge depth of 0.05 mm and a spindle tilt of 2.5 degrees. Based on previous studies [1,24,31], the rotation speed of 600 rpm was fixed, while the welding speeds were, respectively, 20, 40, and 60 mm/min.

### 2.2. Experimental Analyses

**Figure 2:**
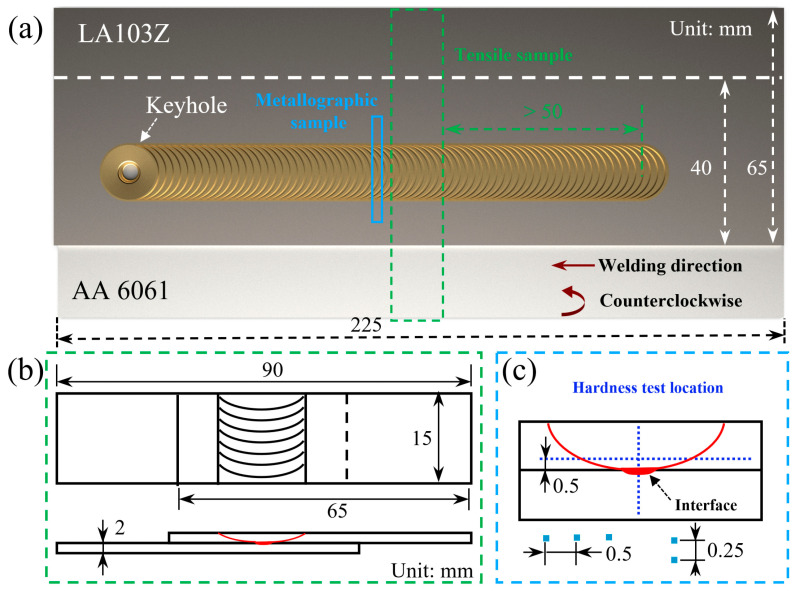
Schematic illustration of (**a**) positions of samplings, (**b**) tensile sample, and (**c**) metallographic sample.

Following welding experiments, metallographic and tensile specimens were both sectioned perpendicular to the welding direction, as shown in Figure 2a. Then, the samples prepared for microstructure examinations were mechanically polished and then etched by the solution (2 mL HNO_3_ + 8 mL H_2_O + 90 mL C_2_H_5_OH) for 5 s. A light microscope (LM, Keyence VHX-1000E, Keyence, Osaka, Japan) and a scanning electron microscope (SEM, SU5000, Hitachi, Tokyo, Japan) equipped with an energy dispersive spectroscopy (EDS, ULTIMATELY MAX40, Oxford Instrument, Oxford, UK) system were employed to characterize the microstructure evolution and elemental distribution. The maximum magnification of the light microscope is 1000X. The sampling depth of the EDS is approximately 1 μm, which can be used to analyze the elements in the range of Be-U. In addition, fracture paths along with the corresponding fracture surface morphologies were examined by LM and SEM. Tensile shear tests were conducted on three specimens using a tensile tester (AG-X plus, Shimadzu, Kyoto, Japan) with a capacity of 20 kN at a crosshead speed of 0.5 mm/min at room temperature. The lap shear strength is characterized by the maximum load of the tensile specimen per unit width. The microhardness distributions were measured perpendicular and parallel to the Mg-Li/Al bonding interface by a microhardness tester (HVS-1000, HuaYin Test Instrument Co., Ltd., Zhengzhou, China) with 200 g weight force and 10 s dwell time. Figure 2b,c provide the dimensions of tensile samples and the target locations of microhardness points.

## 3. Results and Discussion

### 3.1. Appearances and Cross-Sectional Morphologies

**Figure 3:**
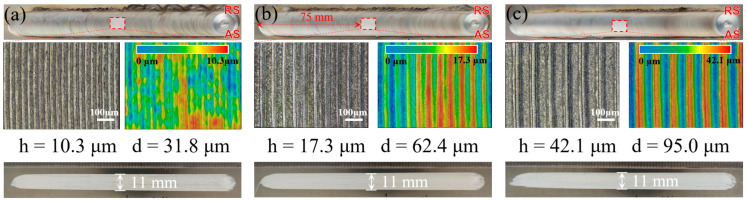
Joint surface appearances at different welding speeds of: (**a**) 20 mm/min, (**b**) 40 mm/min, and (**c**) 60 mm/min. (AS: advancing side, RS: retreating side).

Figure 3 shows the obverse and reverse appearances of the DSFSLW welds. Weld flashes are observed on the retreating side (RS). The formation of the onion skin on the top surface is direct evidence of periodical variation in plastic flow, as studied by Hu [32]. Magnified images of the onion skin reveal that the arc patterns of different joints all exhibit a well periodicity. The intervals (d) between neighboring arc corrugation are 31.8 μm, 62.4 μm, and 95 μm, approximately equal to the distance traveled by the welding tool during one week of rotation. Similarly, the heights (h) of the arc corrugation increase from 10.3 μm to 42.1 μm with increasing welding speed. Smooth scratches, measuring 11 mm in width, are observed on the reverse side of the DSFSLW welds. The axial force generated by the welding tool is mainly supported by the DS-shoulder. Compared with the conventional backing plate, the small size of the DS-shoulder results in a large frictional force between the bottom Al plate and the DS-shoulder during their relative movement, leading to the formation of the scratch morphology.

**Figure 4:**
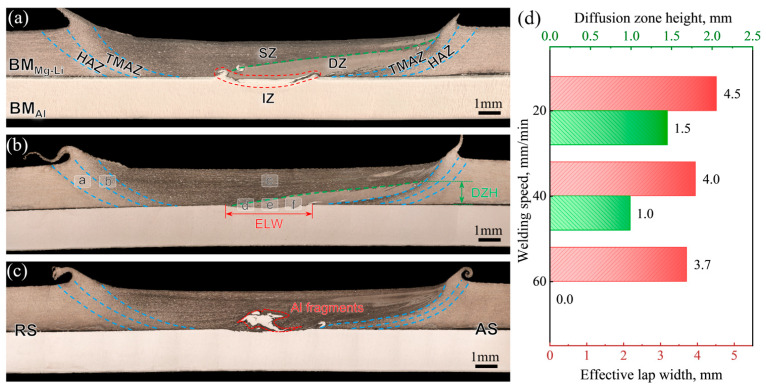
Cross-sectional photographs of joints obtained at different welding speeds: (**a**) 20 mm/min, (**b**) 40 mm/min, (**c**) 60 mm/min, (**d**) DZHs and ELWs.

Figure 4 displays the cross-sectional photographs of the Mg-Li/Al joints obtained at different welding speeds. During the DSFSLW, the material undergoes different welding thermal cycles and stirring effects and, therefore, exhibits different microstructure evolution characteristics. According to the grain morphology, the joint can be divided into basal material (BM), heat affected zone (HAZ), thermo-mechanically affected zone (TMAZ), stir zone (SZ), and interface zone (IZ), as shown in Figure 4a. Among them, the SZ is affected by the tool geometry and mainly shows a “basin” shape. At the bottom of the SZ, a light-colored zone extending from the interface to the advancing side (AS) is observed. This may be caused by the bottom Al entering the SZ driven by the stirring pin and dissolving into the Mg-Li matrix at high temperatures. The diffusion of Al promotes the precipitation of α-Mg phases and inhibits the growth of β-Li phases in Mg-Li alloys while improving the corrosion resistance [33,34]. Visually, the diffusion zone (DZ) appears lighter under OM than the non-diffusion zone (NDZ), as denoted in Figure 4a. The diffusion zone height (DZH) and the effective lap width (ELW) can characterize the degree of plasticization and mixing during DSFSLW, which determines the microstructure and mechanical properties of the joint [23]. Figure 4d presents the DZHs and ELWs of the cross-sections at different welding speeds. With the increase in welding speed, DZHs and ELWs decrease to 200 μm, 100 μm, and 0 μm, as well as 4.5 mm, 4.0 mm, and 3.7 mm, respectively. The varying DZHs and ELWs observed are attributed to the enhancement of the material flow and element diffusion during welding. It is worth noting that when the welding speed increases to 60 mm/min, the diffusion zone basically disappears and is replaced by blocky Al fragments.

### 3.2. Microstructure Features

**Figure 5:**
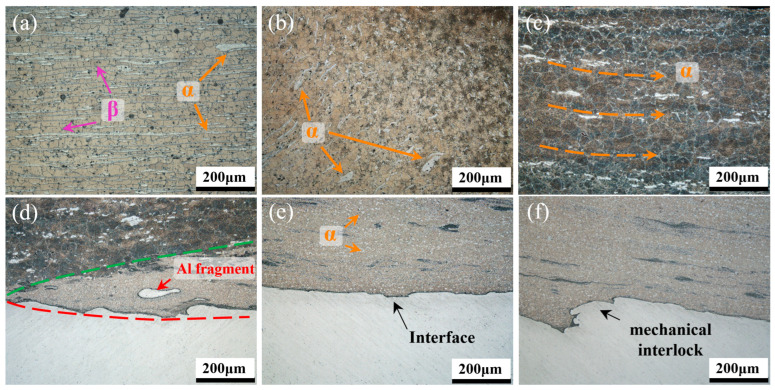
Metallographs of different areas marked in Figure 4: (**a**) HAZ, (**b**) TMAZ, (**c**) SZ, (**d**) IZ-RS, (**e**) IZ-middle, and (**f**) IZ-AS.

Figure 5 shows high-magnification metallographic images that reflect detailed information about the microstructure marked in Figure 4b. The HAZ is only affected by thermal cycles during welding. Therefore, the α-Mg phase maintains the rolling strip shape, and the β-Li phase is distributed between adjacent strip layers with blocky grains. Compared with the HAZ, the TMAZ is located closer to the stirring tool and undergoes higher temperature cycling and more plastic deformation. As a result, the original strip-shaped morphology of the α-Mg phase changes significantly. Some of the α-Mg phases agglomerate and transform into coarse island-like structures, while others are significantly distorted by the complex stress field and transform into short rod-like structures. In the SZ, the α-Mg phase is wholly fragmented and partially dissolved in the matrix due to severe deformation. The remaining α-phase forms island-chain-like structures along the direction of the stirring tool’s rotation. Large equiaxed β-Li-phase grains formed through dynamic recrystallization are observed in the SZ. This can be attributed to the low welding speed and the less heat required for recrystallization and grain growth of β-Li phases compared with α-Mg phases. In comparison to the flat interface in the IZ-middle, clear mechanical locking structures are visible on both sides of the IZ, as depicted in Figure 5d–f. The DZ is separated from the NDZ by a clear boundary that gradually expands from IZ-RS. Additionally, Al fragments that have just detached from the bottom plate are detected. The dual-phase structure is greatly refined for α-Mg phases, which are smashed into dispersed particles and homogeneously distributed among the β-Li phases.

**Figure 6:**
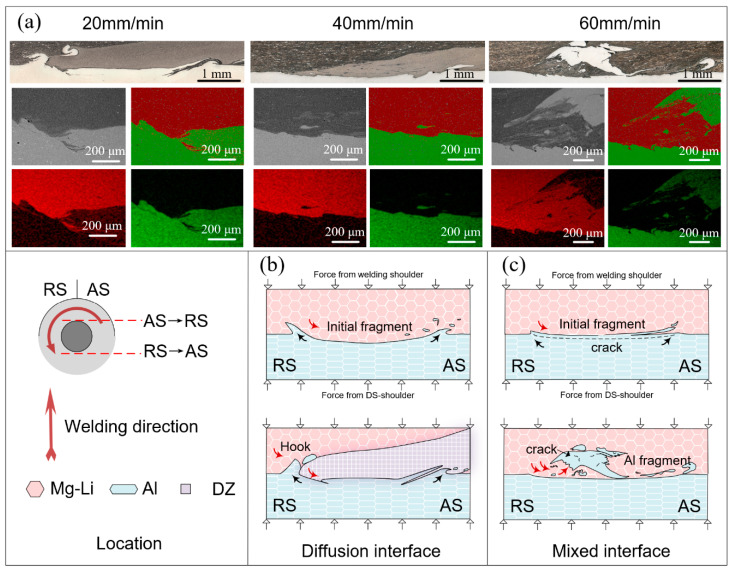
Schematic illustration for the Mg-Li/Al interface evolution: (**a**) morphology of IZs, (**b**) diffusion interface, and (**c**) mixed interface.

The evolution of the joint interface is depicted in Figure 6. The morphology of the interface, distribution of Al fragments, and size of the DZ are markedly influenced by the heat input. At a welding speed of 20 mm/min, the Al alloy exhibits pronounced deformation, resulting in the formation of hook structures on both sides of the IZ. An evident interdiffusion region adjacent to the interlocking structures is observed. Increasing the welding speed diminishes the deformation of Al alloy and the size of DZ. Some Al fragments, not entirely dissolved within the Mg-Li alloy matrix, are present within the DZ. As the welding speed increases to 60 mm/min, the penetration of Mg-Li into the Al matrix is further reduced, and the DZ essentially disappears, supplanted by blocky Al fragments that have just detached. Small, partially dissolved Al fragments are observed around the large Al fragments.

According to the above interface structure characteristics, two types of Mg-Li/Al interfaces, namely the diffusion interface and the mixed interface, can be identified under different welding conditions. As shown in Figure 6b, diffusion interfaces form within joints formed under high heat input conditions. The Al matrix at the base is extruded to both sides by the stirring pin, resulting in the formation of hook structures. A portion of the Al, peeling off from the matrix, is propelled by the plasticized Mg-Li flow, bypassing the stirring pin towards the RS. Under the confluence of elevated temperature and stress, the small Al fragments are further fragmented and diffused, ultimately becoming dissolved within the Mg-Li matrix. The dissolved Al atoms facilitate the re-precipitation of the α-Mg phase and impede the growth of the β-Li phase, thereby resulting in the formation of DZ. As the welding tool rotates, the plastic material continues to flow from RS to AS, filling the instantaneous cavity formed during the forward process of the stirring pin. The hook structure is fractured by the impact of plastic material during this process, forming twisted Al fragments. Concurrently, the threads on the surface of the stirring pin promote the downward flow and deeper penetration of the Mg-Li alloy into the significantly softened Al alloy. This interaction results in the formation of mechanical interlocks and extends the length of the interface connection. For the mixed interface (Figure 6c), the flow stress of the Al alloy increases as the heat input decreases. The Al in direct contact with the stirring pin is broken under the severe shear force and then gradually peels off from the matrix. Then, the turbulent plastic flows, induced by the large Al fragments, accelerate the delamination of Al fragments. Finally, the complex stress–strain field adjacent to the interface leads to further fragmentation of blocky Al fragments, and the exfoliated small fragments are partially dissolved within the Mg-Li alloy matrix under the thermal effect.

**Figure 7:**
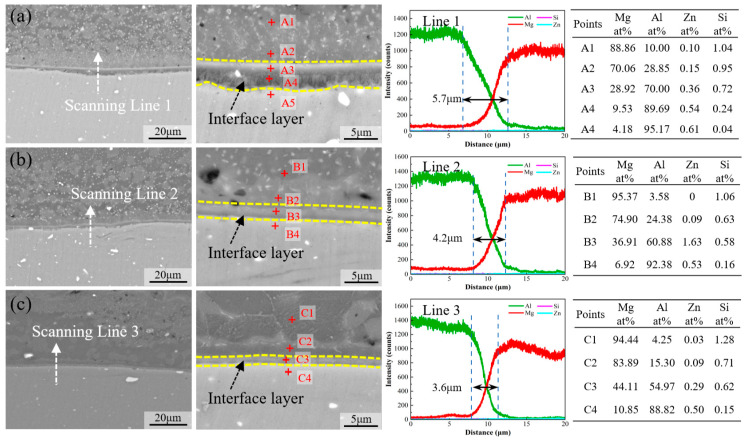
Interfacial characteristics of joints under different welding conditions: (**a**) 20 mm/min, (**b**) 40 mm/min, and (**c**) 60 mm/min.

The SEM and EDS results are presented in Figure 7 to elucidate the detailed interfacial characteristics of different Mg-Li/Al joints. Clear stratification is observed across all three interfaces, with the darker region indicating the Mg-Li alloy and the lighter region indicating the Al alloy, separated by a gray interface layer, suggesting the formation of IMCs during DSFSLW. At the welding speed of 20 mm/min, the interface layer with uneven thickness is observed. The Al alloy near the interface is sufficiently softened and intermixed with the interface layer, influenced by complex strains and heat. In contrast, at speeds of 40 mm/min and 60 mm/min, the interface layer appears more uniform and devoid of intermixing, as shown in Figure 7b,c.

As the Li element is not detectable by EDS, only the element diffusion of Mg, Al, Si, and Zn at the interface has been characterized. With decreasing welding speed, the heat input and interface reaction duration increase, and the interaction between Mg-Li and Al intensifies. Consequently, the thickness of the interface layer increases progressively from 3.6 μm to 5.7 μm. Subsequently, EDS point analysis is utilized to further ascertain the chemical composition adjacent to the interface layer.

In joints welded at lower speeds, the extent and depth of diffusion of Al atoms are markedly greater than in those welded at higher speeds (Points A2, A5, B2, B4, C2, C4). It could be concluded that the diffusion rate of Al atoms within the Mg-Li alloy substantially exceeds that of Mg atoms in the Al matrix, which explains the formation of DZ. No obvious enrichment of Si and Zn elements is detected within the interface layer. A continuous distribution of Al and Mg elements can be observed. Furthermore, EDS analysis within the interface layer corroborates this transitional distribution (Points A2 and A3). Meanwhile, Points A3, B3, and C3 provide the chemical compositions of the interface layers adjacent to the Mg-Li matrix across different joints. As the welding speed increases, the Al content within the interface layer diminishes while the Mg content augments. These variations in elemental distribution, both within the interface layer and among different layers, underscore the complexity of the Mg-Li/Al interface layer’s composition. The liquidus surface of the Al-Li-Mg system shows [35] that a eutectic reaction exists at 418 °C: $L\to {\left(Mg\right)+Al}_{12}{Mg}_{17}+AlLi$. The formation and mixture of Al_12_Mg_17_ and AlLi phases at the interface may account for the transitional distribution of the Al and Mg elements. Nonetheless, the addition of the Li element in the Mg-Li alloy amplifies the complexity of the interfacial reactions relative to conventional Mg alloys. Hence, the specific types of IMCs present at the Mg-Li/Al interface necessitate further experimental and theoretical examination.

### 3.3. Microhardness Distribution

**Figure 8:**
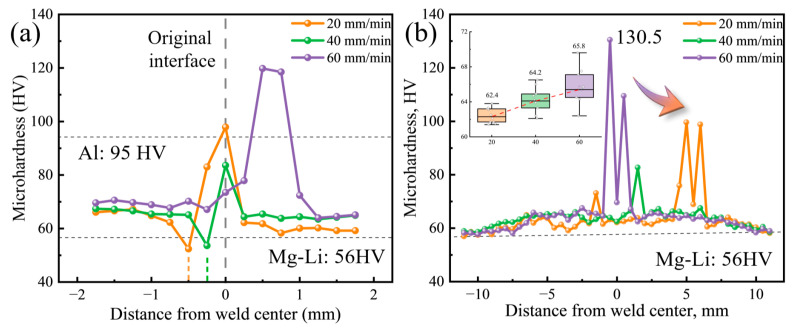
Microhardness distributions of the joints: (**a**) perpendicular to interface and (**b**) parallel to interface.

Figure 8 presents the microhardness distributions across the joints. The microhardness values of the Mg-Li and Al alloy BMs are 95 HV and 56 HV, respectively. In the case of those perpendicular to the interface, the microhardness on the Mg-Li side is slightly increased, whereas the Al side decreases sharply to 56–66 HV. As the welding speed decreases, the depth at which the lowest microhardness value is observed increases, which is consistent with the microstructure. The reduction in microhardness of the Al alloy could be attributed to the dissolution and growth of precipitates during the thermal cycling [36]. High microhardness values, all exceeding 80 HV, were detected within various joints, with the maximum value reaching up to 120 HV. For diffusion interfaces, the high microhardness region appears near the IMCs formed at the interface. For mixed interfaces, the extremely high microhardness value (130.5 HV) derives from the blocky Al fragments inside the SZ. Blocky Al fragments undergo significant strain hardening due to the severe plastic deformation and react with the Mg-Li through atomic diffusion to form IMCs. The combined effect of these interactions promotes the increase in the microhardness value.

In the case of those parallel to the interface, the increase in microhardness of Mg-Li in the SZ, TMAZ, and HAZ is more clearly observed. For Mg-Li alloys, the enhancement of mechanical properties through friction stir processing is a well-documented phenomenon [37,38,39]. Solid solution strengthening and dislocation strengthening are the primary contributors to the increased microhardness in the SZ. High heat input during the process reduces the volume fraction of the α-Mg phase. More Mg atoms dissolve into the Li matrix, resulting in solid solution strengthening. In addition, the material in the SZ undergoes severe plastic deformation and significant work hardening, which also results in an increase in microhardness. These combined effects of heat input and plastic deformation explain the observed increase in microhardness in the SZ. The thermo-mechanical effects in TMAZ and HAZ are diminished, so the strengthening effect is reduced. The embedded box plot shows microhardness statistical data within the SZ after deducting the influence of Al fragments. With the decrease in welding speed, the average microhardness decreased from 65.8 HV to 62.4 HV. This is primarily due to the increased welding heat input, which accelerates the growth of recrystallized grains, reducing the strengthening effect of grain refinement. The low welding speed also prolongs the flow time of the plasticized materials, which in turn increases the moving distance of Al fragments, resulting in the migration of the high microhardness zone towards the AS.

### 3.4. Tensile Shear Test and Fracture Analysis

**Figure 9:**
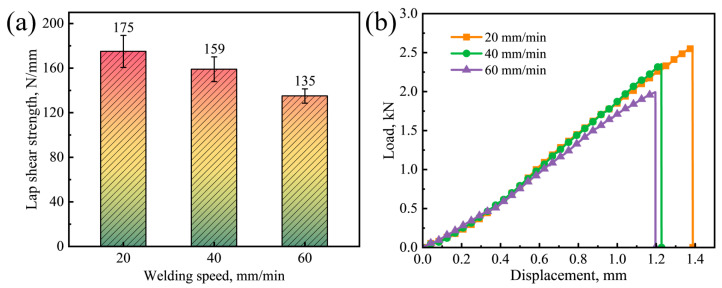
Tensile shear test results of Mg-Li/Al joints: (**a**) shear failure strengths and (**b**) load–displacement curves.

Lap shear tests are conducted to assess the performance of the Mg-Li/Al joints, and the failure strength and load–displacement curves are shown in Figure 9. The tensile strength of as-received LA103Z and 6061-T6 plates is 184 MPa and 304 MPa, respectively, and the equivalent maximum load per unit width is 368 N/mm and 608 N/mm. The failure strength of the joints reaches the maximum of 175 N/mm at 20 mm/min and decreases to 135 N/mm at 60 mm/min. It could be attributed to the insufficient bonding between the Mg-Li and Al alloys. At low welding speed, adequate reaction time and high welding temperature enhanced intermetallic reactions and strengthened the interfacial bonding. The improved interface bonding results in greater deformation, as evidenced by increased elongation. In general, excessive IMCs can induce elevated residual stress within the joint, leading to reduced failure strength. When the thickness of the IMCs layer is less than 10 μm, the interface strength of the FSLW joint is higher [40,41]. Xu et al. [22] observed a 67.9 μm thick eutectic layer at the Mg interface, comprising Mg solid solution and Al_12_Mg_17_ phase. Compared with AZ31 Mg alloys, the heat generated by the Mg-Li alloy during welding is lower, consequently retarding IMC formation [42,43]. Even at a low welding speed of 20 mm/min, the thickness of the IMCs remains below 10 μm. Consequently, an increase in the interface thickness correlates with a higher maximum load of the joints.

**Figure 10:**
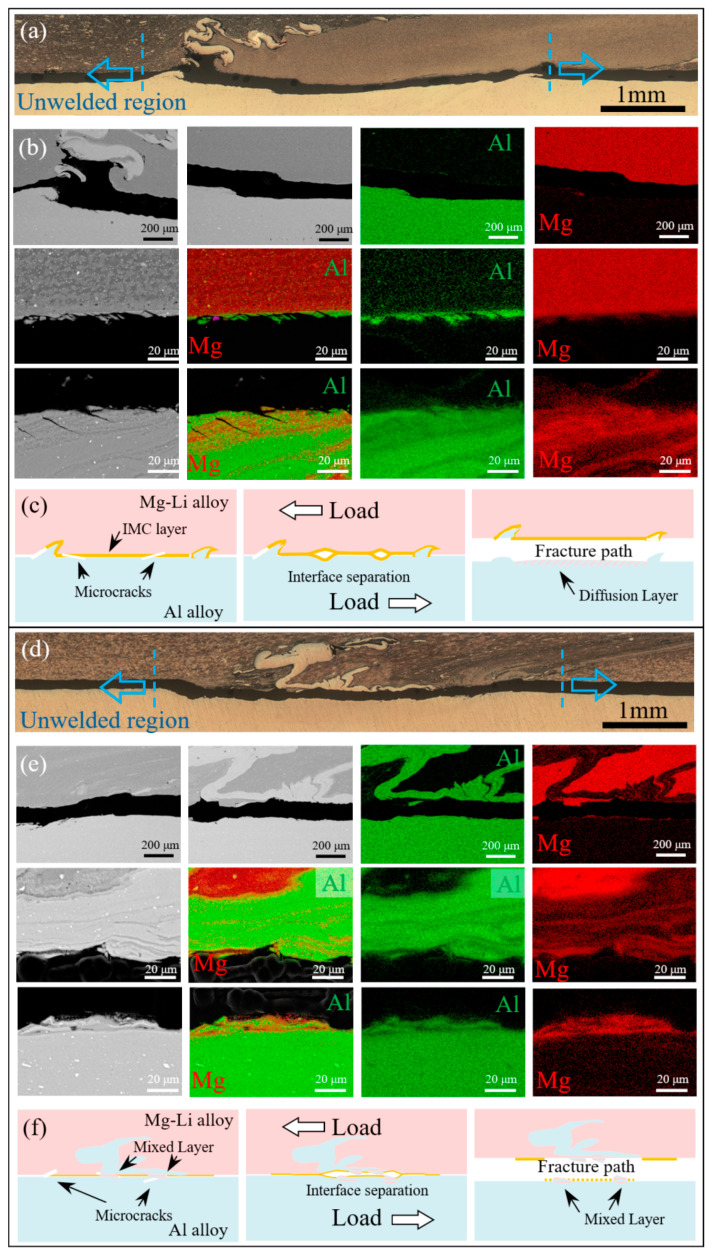
Fracture paths of two representative interface specimens: (**a**) overall morphology at 20 mm/min, (**b**) enlarged views and corresponding EDS mapping from (a), (**c**) schematic of the diffusion interface fracture path, (**d**) overall morphology at 60 mm/min, (**e**) enlarged views and corresponding EDS mapping from (d), and (**f**) schematic of the mixed interface fracture path.

Following the tensile shear tests, the fracture paths of two representative interface specimens are depicted in Figure 10. All specimens were fractured along the Mg-Li/Al interface, each with distinct mechanisms. For the diffusion interface, the propagation of cracks initiated in the unwelded areas on both sides of the IZ is impeded by the hook structure, thus changing the fracture path. Furthermore, EDS analysis reveals a distinct aluminum-rich layer on the Mg-Li side, which can be identified as the remnant of the fractured IMCs. Numerous secondary tearing cracks are also observed on the Al side. This indicates that a high degree of interfacial bonding, facilitated by high temperatures, has enhanced the interaction between Mg-Li and Al. Nonetheless, the considerable property disparity between the dissimilar materials results in substantial residual stresses at the interface upon welding. Moreover, given the brittle nature of IMCs, significant stress concentration occurs during tensile shear experimentation. Owing to the exceptional plasticity of the dual-phase Mg-Li alloy (over 40%), it can more effectively accommodate the deformation discrepancy relative to the IMCs. Conversely, the Al side, with its lower plasticity in conjunction with stress concentration and lattice mismatch, initiates microcracks at the IMC/Al interface. Subsequently, the cracks propagate along the IMC layer and converge with the cracks at the root of the hook structure, ultimately leading to the joint’s failure.

However, for the mixed interface, the absence of the hook structure enables the cracks initiated in the unwelded areas on both sides of the IZ to expand straight. SEM and EDS analysis results reveal that the fracture propagates along the mixture of Mg-Li, Al, and Al fragments. Layered Mg-Li/Al mixtures are observed on both sides of the fracture path. Discontinuous IMCs form through atomic diffusion between Al fractures and Mg-Li lamellas. The blocky Al fragments impeded the vertical flow of the Mg-Li alloy, thereby reducing the hydrostatic pressure at the interface. Thus, the interfacial reaction rate is diminished [44]. Coupled with the lower heat input at high welding speeds, the interfacial reaction duration is abbreviated, resulting in inadequate interfacial reaction and diminished bond strength.

**Figure 11:**
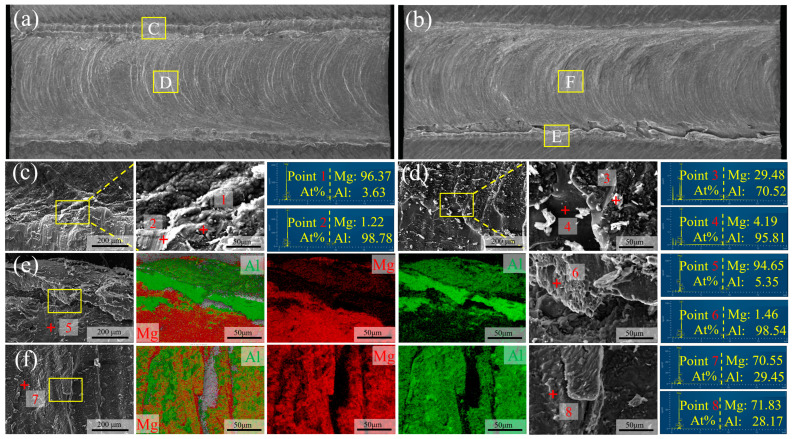
Fracture surfaces of the failed specimens for 20 mm/min: (**a**,**c**,**d**) Al side; (**b**,**e**,**f**) Mg-Li side.

The fracture surface morphologies at different positions of typical joints are examined to ascertain the effect of interfacial microstructure on the fracture behavior. Figure 11a,b depict the fracture morphologies of Al and Mg-Li sides, respectively, for the joint welded at 20 mm/min. The fracture surface exhibits an onion skin morphology akin to that of the top surface, indicative of the interface having undergone vigorous stirring. Notably, hook structures are observed on the Al side, and corresponding cavities are observed on the Mg-Li side, with the enlarged views presented in Figure 11c,e. On the Mg-Li side, fractured layered materials, composed of more than 98% Al alloy, are observed adjacent to the cavities. Prominent dimples are observed within the layered Al region, demonstrating high plasticity and typical ductile fracture characteristics. These observations suggest that the hook structure serves as an effective load bearing to improve the strength of the joint during the shear tests, which correlates well with the stress–strain curve in Figure 9. As illustrated in Figure 11d,f, the distinct layered characteristics at the center of the fracture indicate the formation of a complex micro-interlocking structure between Al and Mg-Li. Furthermore, an abundance of interfacial IMC particles is present on the Al side, which approximates the fracture surface on the Mg-Li side. Combined with the EDS results, this suggests that the fracture is more likely to propagate through the interface and the Al matrix than the Mg-Li matrix.

**Figure 12:**
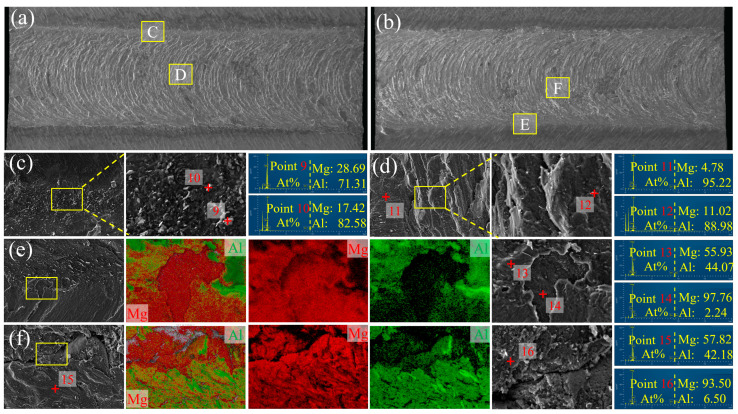
Fracture surfaces of the failed specimens for 60 mm/min: (**a**,**c**,**d**) Al side; (**b**,**e**,**f**) Mg-Li side.

Figure 12a,b depict the fracture morphologies of Al and Mg-Li sides for the joint welded at 60 mm/min. The onion skin morphology is also evident, while hook structure is not apparent on either side of the fracture surface. Instead, a flat surface is present. The distribution of IMC particles and the fragmentation of the IMC layer on the Mg-Li side (as illustrated in Figure 12c,e,f) indicate that the IMC layer’s adhesive strength to the matrix is compromised. This could be due to the lower heat input at higher welding speeds, which limits the extent of interfacial reactions necessary for a robust bond. The exposure of the Mg-Li alloy matrix in the central region of the fracture surface provides further evidence to support this hypothesis, suggesting that the fracture is more likely to initiate at the interface or within the Al matrix, where the IMC layer possesses a low adhesive strength to the matrix.

## 4. Conclusions

In this work, LA103Z Mg-Li and 6061 Al alloy joints were fabricated by dynamic support friction stir lap welding (DSFSLW). The joint formation, interfacial characteristics, and fracture behavior of Mg-Li/Al joints were studied. The main conclusions are as follows:

1. Two types of interfaces have been delineated to elucidate the interface formation: the mixed and the diffusion interfaces. Diffusion interfaces involve the fragmentation and dissolution of Al fragments into the Mg-Li matrix, which in turn promote α-Mg phase re-precipitation and the formation of a diffusion zone. Mixed interfaces, associated with reduced heat input, exhibit increased Al flow stress, leading to broken Al peeling off and turbulent plastic flows, influencing interface bonding.
2. The welding speed exerts a significant influence on the interaction, with slower speeds enhancing the interfacial reaction and leading to a thicker interface layer, ranging from 3.6 μm to 5.7 μm. Intense material flow further enhances mechanical interlocking and interface connection length.
3. The microhardness on the Mg-Li side is slightly increased, while the Al side experiences a decrease. Extremely high hardness values within the joints are ascribed to the formation of IMCs and strain hardening of blocky Al fragments.
4. The fracture behavior confirms the complex relationship between welding conditions, microstructure, and mechanical performance. All specimens fractured unevenly along the Mg-Li/Al interface due to the IMC formation. The maximum failure strength of 175 N/mm was achieved at 20 mm/min, aligning with the assumption that lower welding speeds would enhance joint strength due to better interfacial reactions.
5. Future work is expected to focus on advanced material characterization and improving welding efficiency. In situ monitoring and phase analysis will provide insight into interface evolution. Additional energy fields will help to expand the optimal conditions for improving mechanical performance, making manufacturing more efficient and cost effective for aerospace and automotive industries.

## Figures and Tables

**Table 1:** Chemical composition of 6061-T6 Al alloy and LA103Z Mg alloy.

Materials	Chemical Composition (wt%)									
	Cu	Mn	Fe	Zn	Cr	Ti	Si	Li	Mg	Al
6061-T6	0.15–0.4	0.15	0.7	0.25	0.04–0.35	0.15	0.4–0.8	/	0.8–1.2	Bal.
LA103Z	<0.01	/	<0.01	3.05	/	/	<0.01	10.16	Bal.	3.01

## Data Availability

The original contributions presented in the study are included in the article, further inquiries can be directed to the corresponding author.
